# How Do Quacks Get Their Address Lists?

**Published:** 1913-11

**Authors:** 


### How Do Quacks Get Their Address Lists?

Have you ever noticed how often you, a physician, receive circulars addressed to you as "Dr." or "M. D.," asking you to use, as a patient or as a prescriber, some nostrum of the worst type?

Have you ever reflected that it might be more than a coincidence that your patients, while confined to bed and under active treatment, receive circulars of this sort?

Have you ever seen a recently bereaved family at the moment when its emotions were being harrowed by a circular of certain cure, received a few days too late?

Have you ever been asked to join in a laugh at the expense of a very respectable, well-to-do invalid, who was offered a chance to earn good money by acting as agent for Old Dr. Curem's Platitudinous Plaster?

We have seen enough illustrations of these conditions to convince us that they are not mere coincidences. There must be enough regularly listed physicians willing to use quack medicines to pay for circularizing the entire profession in certain localities. And there must be a regular system of reporting invalids, persons with the quack medicine habit, and seeking men and especially women of more or less leisure and influence, who would yet like to add to their incomes by easy means. Just what the system is we do not know. We do know that lists of names have a relatively high commercial value; also that there are many advertisements of easy work which does not require salesmanship and which may be done without interfering with other duties. We suspect that, somehow, there is being maintained a pretty thorough system of espionage over the health of the population, with a view to putting the manufacturers of quack medicines on the right track.

